# Perspectives of US Youths on Participation of Transgender Individuals in Competitive Sports: A Qualitative Study

**DOI:** 10.1001/jamanetworkopen.2022.55107

**Published:** 2023-02-08

**Authors:** Alexander Waselewski, Marika Waselewski, Eric Waselewski, Laura Kruger, Tammy Chang

**Affiliations:** 1 Department of Pediatrics, University of Michigan, Ann Arbor; 2 Department of Family Medicine, University of Michigan, Ann Arbor; 3 Department of Internal Medicine, University of Michigan, Ann Arbor; 4 Institute for Healthcare Policy and Innovation, University of Michigan, Ann Arbor

## Abstract

**Importance:**

Rights and access for transgender individuals, including the participation of transgender athletes in sports, have long been debated. These discussions often center around fairness and mental health impacts on youths associated with identity-based inclusion in sports.

**Objective:**

To assess the experiences and perspectives of adolescents and young adults on the inclusion of transgender individuals in competitive sports.

**Design, Setting, and Participants:**

In this qualitative study, 5 open-ended survey questions were sent to the MyVoice cohort from December 10 to 17, 2021. MyVoice is a nationwide text-message polling platform of US youths aged 14 to 24 years. All coding and subsequent analysis was completed between January 10 and December 11, 2022.

**Main Outcomes and Measures:**

Qualitative perspectives of youths regarding transgender athlete participation in sports as measured by survey responses. Responses were reviewed using an inductive approach to qualitative thematic analysis to develop a codebook. The codes were independently applied to all responses by 2 investigators; discrepancies were resolved with discussion. Summary statistics were calculated for demographic characteristics and code frequencies, and χ^2^ tests (α = .05, 2-tailed) were used to evaluate differences in opinion based on gender identity and participation in competitive sports.

**Results:**

A total of 905 of 1199 youths (75%) responded to the survey. Respondents had a mean (SD) age of 20 (2) years; 482 (53%) identified as male, 29 (3%) identified as transgender, and 306 (34%) reported having participated in high school and/or collegiate athletics. Three themes emerged: (1) youths differed regarding the inclusion of transgender athletes based on gender identity vs sex assigned at birth, (2) many youths did not have personal experience related to the inclusion of transgender athletes, and (3) youths were uncertain about the impacts of gender identity–based participation on cisgender individuals but perceived positive impacts for transgender individuals. Nearly half of respondents (327 of 691 [47%]) thought that transgender athletes should participate based on their gender identity or personal preference, whereas 240 (35%) favored participation based on sex assigned at birth or in a transgender-only category. Respondents mentioned concern about the fairness of identity-based participation, specifically for cisgender women, but many (410 of 697 [59%]) also reported that it would be affirming for transgender athletes to participate based on gender identity.

**Conclusions and Relevance:**

The youths in our study differed in their opinions regarding sports participation of transgender youths, but many felt that inclusive policies would affirm and support the mental health of transgender individuals. Negative impacts on fairness were noted by some respondents. These findings suggest that nuanced policies are needed to address the participation of transgender athletes in competitive sports and should consider the impacts on and perspectives of youths most affected.

## Key Points

**Question:**

What are the opinions of US youths on the participation of transgender individuals in competitive sports?

**Findings:**

In this qualitative study of 905 adolescents and young adults, 47% felt that transgender athletes should be able to participate in competitive sports based on gender identity, 35% felt that participation should be based on sex assigned at birth, and 10% stated that participation should be based on the stage of transition, hormone levels, or sport being played.

**Meaning:**

The diverse responses of individuals who responded to the study questions suggest that policy makers should consider the perspective of youths and the associated impacts when developing guidelines on the participation of transgender athletes in competitive sports.

## Teaser

This qualitative study surveys adolescents and young adults in the US to assess their experiences and perspectives on the inclusion of transgender individuals in competitive sports.

## Introduction

Transgender individuals have been the focus of numerous discussions across all areas of life. What they can and cannot do and where they can and cannot go is frequently litigated at both the state and national levels in the US. While no federal law prohibits sex- or gender identity–based discrimination in public accommodations, most states have laws prohibiting sex-based discrimination.^1^ Some states have additional laws specifically protecting gender identity– or gender expression–related discrimination in employment, housing, and schools.^2,3^ However, multiple bills restricting transgender rights have recently been passed in the US, and discussion of these efforts includes inflammatory statements such as comparing transgender youths to “terrorists.”^4^ Some states have enacted laws that (1) prohibit accommodations for restrooms or changing rooms for transgender youths that align with their gender identity^5^ and (2) allow students to seek legal action if they believe they shared a restroom with a student of the opposite sex.

Beliefs surrounding transgender individuals have also led to debate regarding the inclusion of transgender athletes in sports based on gender identity or sex assigned at birth. Legislation has been proposed or passed in 32 states banning transgender athletes from competing based on their gender identity in school sports.^6^ Some of these state laws are being challenged and brought to federal court.^7^ Laws requiring individuals to participate based on their sex assigned at birth are based on concerns of fairness in competition.^8^ The success of some transgender women in sports has generated negative attention from both teammates and outsiders related to the biological advantages of male individuals, such as higher lean body mass and muscle.^9,10,11^

Conversely, laws and consensus statements allowing or encouraging transgender individuals to compete based on their gender identity are becoming more prominent. Seventeen states plus Washington, DC, now have inclusive policies regarding transgender athletes in schools.^12,13^ The International Olympic Committee^14^ also recently released a statement that set guidelines on how individual sports of the highest competition level should determine inclusion of transgender athletes and recommended against hormone level–based inclusion, but ultimately left the decision to the governing body of a given sport.

National organizations such as the American Academy of Pediatrics^15^ and the American Medical Association^16^ have also released statements opposing legislation that prevents transgender youths from participating in society as their identified gender, due to impacts on health and well-being. Transgender youths experience mental health disorders—including depression, anxiety, self-harm, and suicide—at higher rates compared with their cisgender peers.^17^ Studies have shown that biological or social affirmation of transgender youths leads to improvements in mental health.^17^ Youth participation in sports results in positive outcomes, both personally and professionally, and exclusion compounds already poor mental health among transgender youths.^18,19,20^

While these debates impact both transgender and cisgender youths, laws and policies on the topic of sports participation among transgender youths are primarily dictated by adults. Concerns about fairness in sports, safety of children, and mental health outcomes are frequently cited without inclusion of the youth perspective. The aim of this study was to assess the experiences and perspectives of adolescents and young adults on the inclusion and impact of transgender individuals in competitive sports.

## Methods

### Study Design and Participants

Our qualitative study used the nationwide MyVoice cohort, a longitudinal text-message polling program that seeks to understand youths’ opinions on salient health and policy issues affecting them. MyVoice represents a diverse sample of youths aged 14 to 24 years from across the US. MyVoice uses text messaging because nearly all youths use texting as a form of communication, making the program highly accessible to this population, including those who cannot or do not typically participate in research. Participants were recruited via targeted social media advertisements based on national benchmarks from weighted samples of the American Community Survey.^21^ Individuals with the following criteria were eligible to participate: age 14 to 24 years, English literacy, and access to a phone with text-messaging capabilities. This study was approved by the University of Michigan Institutional Review Board with a waiver of parental consent for minors. Written informed consent and/or assent was obtained online for all participants.^22^ Demographic information, including self-reported age, gender, race and ethnicity, education, and US region, was collected at study enrollment. This study followed the Standards for Reporting Qualitative Research (SRQR) reporting guideline. In addition, MyVoice is a member of the American Association for Public Opinion Research (AAPOR) Transparency Initiative, hence the study adhered to their guidelines for survey research.^23,24^

Five open-ended questions on respondent participation in sports, effects of transgender individuals in sports, and opinions on the inclusion of transgender individuals in sports were sent to MyVoice participants on December 10 and 13, 2021. Open-ended questions were used to allow youths to share their thoughts in a narrative and unstructured way to get at the nuances of their lived experiences. The questions were developed iteratively and analyzed by a team of youths and researchers with methodologic and adolescent health expertise, resulting in the following language:

1. Did you compete in competitive sports in high school or college (eg, school team, AAU [Amateur Athletic Union], travel teams, etc)? Tell us about it.

2. Do you think transgender people should play competitive sports based on their gender identity or their sex assigned at birth? Why?

3. Do you know anyone who wasn’t allowed to play sports due to their gender identity? What happened?

4. How do you think it impacts cisgender (not transgender) people if trans people are allowed to play competitive sports based on their gender identity?

5. How do you think it impacts trans people if they are allowed to play competitive sports based on their gender identity?

Data collection ended on December 17, 2021.

### Statistical Analysis

Survey responses were downloaded at the end of the data collection period, deidentified, and merged with respondent demographic data using unique study identifiers. Survey responses were reviewed by 4 authors (A.W., M.W., E.W., and L.K.) using an inductive approach to qualitative thematic analysis to develop a codebook. All responses were then coded independently by pairs of these 4 authors, with discrepancies resolved through discussion. Overarching themes were then identified and discussed among the authors. All 4 coding investigators are aged between 25 and 30 years and identify as cisgender. Three (A.W., E.W., and L.K.) are physicians in the fields of pediatric endocrinology, sports medicine, and family medicine, respectively, and have all provided medical care to transgender individuals and student-athletes. All of the investigators had no relationship to participants and no personal attributes or experiences that may have influenced the research.

Summary statistics for self-reported demographic characteristics and code frequencies, excluding nonsensical responses, were obtained with Excel 2016 (Microsoft Inc). Opinions on transgender inclusion in sports were compared among individuals who did and did not play competitive sports and across gender identities using the χ^2^ test (α = .05, 2-tailed). Statistical analysis was performed on December 11, 2022, using SAS, version 9.4 (SAS Institute Inc).

## Results

Of the 1199 MyVoice participants surveyed, 905 (75%) responded to at least 1 question. A median of 5 questions (range, 1-5) were answered per respondent. The mean (SD) age of the respondents was 20 (2) years. A total of 482 respondents (53%) identified as male, 333 (37%) as female, 29 (3%) as transgender, and 61 (7%) as other (Table 1). With regard to race and ethnicity, 108 (12%) respondents self-identified as Asian, 91 (10%) as Hispanic, 60 (7%) as non-Hispanic Black, 589 (65%) as non-Hispanic White, and 55 (6%) as other (including American Indian or Alaska Native, Native Hawaiian or other Pacific Islander, multiple races or ethnicities, or other race or ethnicity). Competitive sporting experience was also assessed in this survey, and just over one-third of respondents (306 of 894 [34%]) reported having played competitive sports. Most youths who indicated they had played sports reported participating in high school (178 [58%]), with 15 (5%) noting participation at the collegiate level (Table 2).

**Table 1.** Demographic Characteristics of Survey Respondents

Characteristic	No. of respondents (%) (N = 905)
Age, mean (SD), y	20 (2)
Gender identity	
Male	482 (53)
Female	333 (37)
Transgender	29 (3)
Other^a^	61 (7)
Race and ethnicity^b^	
Asian	108 (12)
Hispanic	91 (10)
Non-Hispanic Black	60 (7)
Non-Hispanic White	589 (65)
Other^c^	55 (6)
Education	
Less than high school^d^	220 (24)
High school graduate	130 (14)
Some college or technical school	380 (42)
Associate’s or technical degree	78 (9)
Bachelor’s degree or higher	97 (11)
US region^e^	
Midwest	271 (30)
Northeast	171 (19)
South	249 (28)
West	213 (24)

^a^ Includes nonbinary and self-reported “other” identity.

^b^ Race and ethnicity are missing for 2 respondents because they did not complete those questions in the demographic survey.

^c^ Includes American Indian or Alaska Native, Native Hawaiian or other Pacific Islander, multiracial, or self-reported race as “other.”

^d^ Includes participants still in high school.

^e^ Region is missing for 1 respondent because they did not complete that question in the demographic survey.

**Table 2.** Questions, Codes, Frequencies, and Sample Quotes

Question and code	No. of respondents (%) (N = 905)^a^	Sample quote
**Did you compete in competitive sports in high school or college (eg, school team, AAU, travel teams, etc)? Tell us about it. (n = 894)**		
No	588 (66)	“No, sadly I didn’t”
		“I have not competed”
Yes	306 (34)	“I did in high school”
		“Yes, travel and club”
Team sports	123 (40)	“Yes I play basketball”
		“Yes I played 4 years of varsity softball”
Individual sports	151 (49)	“Yes I did gymnastics”
		“Yes, I competed in track”
Contact sports	80 (26)	“Yes, I wrestled”
		“Yes basketball”
Noncontact sports	151 (49)	“Yes, I played volleyball in school”
		“yes, i did swim”
High school level	178 (58)	“I was on my freshmen volleyball team in high school!”
Collegiate level	15 (5)	“college student athlete”
**Do you think transgender people should play competitive sports based on their gender identity or their sex assigned at birth? Why? (n = 691)**		
Nonrestrictive policies (gender identity or individual preference)	327 (47)	“I think they should play on whatever team they want to”
		“Let’s not divide sport teams based on sex or gender identity in general”
Restrictive policies (sex assigned at birth or separate league)	240 (35)	“Their sex assigned at birth because that way they’re competing with similar bodied individuals”
		“I don’t think they should be allowed to play sports at all”
Depends	72 (10)	“I think it depends on the stage of their transition. Someone may identify as trans but not use any hormone therapy…”
		“I think each case is different”
Unsure	43 (6)	“I’m not sure both sides have good arguments”
Reason for preferring restrictive policies		
Fairness	183 (26)	“Their sex assigned at birth because that way they're competing with similar bodied individuals”
		“Sex assigned at birth. That’s only fair”
Cis-female disadvantage	66 (36)	“Sex assigned at birth because men naturally are more athletic and it wouldn’t be fair for the women competing”
Biological differences	120 (17)	“it’s hard for a female to be on the same competitive level as a biological male due to muscle composition despite artificial or natural hormone levels”
Reason for preferring nonrestrictive policies		
Inclusivity	95 (14)	“Their gender identity because making them compete otherwise is very disrespectful”
		“because not being allowed to can cause gender dysphoria”
It is “who they are”	75 (11)	“I think…because it doesn’t really affect anything other than how accepted the person feels”
		“Gender identity because that’s who they are”
Biological sex not that important	58 (8)	“because it doesn’t really affect anything other than how accepted the person feels”
		“Most trans people do not have an overall advantage in their sport”
**Do you know anyone who wasn’t allowed to play sports due to their gender identity? What happened? (n = 772)**		
No	730 (95)	“actually no I’ve never met anyone with that problem”
Not personally	59 (8)	“No, not personally”
		“On the news. Its [sic] kind of sad. It should be case by case basis”
Yes	35 (5)	“i know some cases”
		“Yes, one of my trans (FtM) friends were…”
Rules or disqualification	12 (34)	“Yes, in ND there are strict rules and people weren’t allowed to play on teams with the preferred gender identity”
Avoided sports	7 (20)	“...until they finally dropped out and attended an alternative school instead”
Bullied or negative mental health impact	4 (11)	“Yes, they almost killed themselves sophomore year”
**How do you think it impacts cisgender (not transgender) people if trans people are allowed to play competitive sports based on their gender identity? (n = 767)**		
No impact	255 (33)	“i don’t think it effects [sic] them at all”
Definite impact	265 (35)	“It would ruin competition by given them an unfair biological advantage”
Possible impact	167 (22)	“I think in some individual sports it can lead to an unfair advantage”
Unsure	75 (10)	“Don’t really know enough about the subject”
Impact type (n = 432)^b^		
Negative		
Fairness	283 (66)	“It impacts cisgender people since it makes things unfair for them”
Cis-female disadvantage	149 (34)	“i don’t think it affects cis males at all, but obviously cis females are put at some disadvantage”
Upsetting or frustrating	49 (11)	“I think it would impact how much they enjoy the sport”
Discomfort	37 (9)	“It would affect in an uncomfortable or negative way”
Financial opportunities	15 (3)	“I think this is an unfair option for cis women, who would risk missing out on sporting opportunities that could change their lives”
Positive		
Improves experiences	13 (3)	“I don’t think it has a large impact. Diversity and inclusion positively impacts everyone involved”
Increases competition	12 (3)	“I think it will affect your training that it should become more intense and sustained, in the case of women”
**How do you think it impacts trans people if they are allowed to play competitive sports based on their gender identity? (n = 765)**		
Definite impact	611 (80)	“I feel like they would feel good because so much of society already shuts them out”
		“I think it makes them feel more accepted and welcomed as the person they are”
Possible impact	86 (11)	“If people were accepting I think it would be affirming…”
		“they could either be at an advantage or disadvantage”
No impact	22 (3)	“I don't think at all”
Impact type (n = 697)^b^		
Positive		
Affirming	410 (59)	“It could boost their confidence and could help them feel like they fit in more”
		“I think it supports them”
Improves mental health	135 (19)	“I think it will greatly reduce dysphoria for individuals…”
		“It could boost their confidence…”
Increases number of athletes	47 (7)	“I think it is positive for…their sports career because they can freely live every competitive experience”
		“I think it can make participation easier for them”
Negative		
Discrimination	63 (9)	“heavy judging”
		“It might lead them to being depressed cos of favoritsm [sic] being practiced”
Unspecified		
Fairness	112 (15)	“false sense of accomplishment”
		“It gives them an advantage that isn’t fair”

Abbreviations: AAU, Amateur Athletic Union; FtM, female to male.

^a^ Percentages were calculated based on question-specific response rates. Percentages may not sum to 100%, as codes are not mutually exclusive, not all codes are presented, and subgroup codes are included.

^b^ Determined by respondents who believed there would be a definite or possible impact.

Three main themes emerged from the review of youths’ perspectives on the inclusion of transgender athletes in sports. First, youths differed regarding the inclusion of transgender athletes based on gender identity vs sex assigned at birth. Second, many youths did not have personal experience related to the inclusion of transgender athletes. Third, youths were uncertain about the impacts of gender identity–based participation on cisgender individuals but perceived positive impacts for transgender individuals. Survey questions, code names and frequencies, and example quotes from participants are provided in Table 2. Discussion of the 3 themes follows.

### Differing Opinions Among Youths Regarding Inclusion of Transgender Athletes Based on Gender Identity vs Sex Assigned at Birth

Of the 691 youths who responded regarding the inclusion of transgender athletes based on gender identity vs sex assigned at birth, 327 (47%) reported that transgender individuals should be allowed to complete based on their gender identity or preference when prompted to choose. However, many respondents (240 [35%]) felt that transgender individuals should participate based on their sex assigned at birth, should participate in transgender-only leagues, or should not be allowed to participate at all. Others were less decisive in their responses (72 [10%]) and felt that participation should be dependent on factors such as the stage of transition, hormone levels, competition level, type of sport (eg, contact vs noncontact), or transition gender. The distribution of opinions based on respondent gender identity (male, female, or other) was significantly different (χ^2^ = 83.99, *df* = 6; *P* < .001), with male individuals (181 of 370 [49%]) being most likely to support restrictive practices compared with female individuals (57 of 242 [24%]) and individuals of other identities (2 of 70 [3%]). These opinions were not significantly different when compared among individuals who did and did not play competitive sports (χ^2^ = 3.17, *df* = 3; *P* = .37).

In explaining their opinions, youths (183 [26%]) most commonly mentioned concern that “it is unfair for the other athletes” to include transgender youths based on gender identity. In particular, 66 (36%) mentioned biological female individuals having a competitive disadvantage (eg, “the physical advantage of transgender women over their female opponents is evident”). Others (120 [17%]) more generally cited biological differences between male and female individuals in their responses. For example, a participant stated that “there are inherent differences biologically that can give trans athletes an advantage over cis athletes.”

Youths noting reasons for nonrestrictive inclusion of transgender athletes most commonly mentioned the importance of inclusivity “because making them compete otherwise is very disrespectful” (95 [14%]) or that gender identity is simply “who they are” (75 [11%]). A few respondents (58 [8%]) also felt that “different levels of hormones are natural in all athletes and can play a role in competition regardless of sex” or that skill rather than gender should divide sports.

### Lack of Personal Experiences Among Youths Regarding Inclusion of Transgender Athletes

Of the 772 individuals who responded regarding their personal experience with inclusion of transgender athletes, 730 (95%) reported not knowing anyone who was kept from playing sports based on their gender identity. Respondents who noted experiences related to restrictive policies for transgender athletes mentioned rules against participation or formal disqualification, bullying or negative effects on mental health, and avoidance of sports in response to exclusion (Table 2). One respondent stated that “one of my trans (FtM) friends were *technically* allowed to play on a men’s football team in high-school, but socially was not; they were outcasted, harassed, and bullied until they finally dropped out and attended an alternative school instead.”

### Uncertainty Among Youths Regarding the Impacts of Gender Identity–Based Participation on Cisgender and Transgender Individuals

There were 767 youths who responded to the question on the impacts of gender identity–based participation on cisgender individuals. When prompted, many youths felt that there might be some impact for cisgender individuals if transgender people played competitive sports based on gender identity, with 265 (35%) noting a definite impact and 167 (22%) noting a possible impact. Another 255 (33%) thought that there would be no impact and 75 (10%) were unsure. Among the 432 youths predicting a definite or possible impact, the types youths mentioned were primarily negative, with respondents citing concerns about fairness (283 [66%]), disadvantages for cis-female athletes (149 [34%]), or upset or frustration for cisgender individuals (49 [11%]). A few also noted that transgender athlete participation may create discomfort (37 [9%]) or affect scholarships or paid sporting opportunities (15 [3%]). One participant noted, “It makes things like scholarships more difficult to accurately give out, I feel. I think cis-females would struggle much more than cis-males in terms of any unfairness.”

When asked about the impacts on transgender individuals, most respondents (611 of 765 [80%]) felt that there would be a definite impact if they were allowed to play competitively based on their gender identity and another 86 ([11%]) felt that there might be an impact. Of the 697 individuals reporting some level of impact, 410 (59%) reported feeling that it would be affirming for transgender athletes to be able to participate based on gender identity and 135 (19%) specifically mentioned that it might improve transgender athletes’ mental health. Some respondents, however, mentioned the potential for negative experiences like discrimination from other athletes or the public if allowed to participate based on gender identity. Impacts related to fairness were noted here as well (112 of 697 respondents [15%]) and referenced both transgender male disadvantage and transgender female advantage in competing by gender identity. One participant stated, “I think it is gender reaffirming but at the same time, trans men will have a disadvantage playing against cis men and trans women have an advantage against cis women.”

## Discussion

Youths in our nationwide qualitative study reported differing opinions on whether they thought transgender individuals should compete in sports based on gender identity or sex assigned at birth. Many participants felt that there would be impacts on both cisgender and transgender athletes with participation based on gender identity. These impacts were mostly noted to be negative for cisgender athletes and positive for transgender athletes. In describing these perspectives, there were recurring concerns about fairness, especially for cisgender women, as well as the desire for affirmation or inclusion of transgender individuals.

Consistent with existing data,^18^ youths in our survey anticipated positive impacts of affirmation and improved mental health for transgender athletes with identity-based participation. Gender affirmation, whether social or physical, improves mental health outcomes in transgender individuals.^17^ Our findings suggest that participation in sports among transgender individuals should be encouraged, as they experience disproportionately high rates of poor mental health.^17^ Additionally, our survey findings and reports of nonparticipation due to discrimination^19,25^ suggest that the number of athletes,^18^ and level of competition, may increase if transgender youths can participate based on gender identity. Less than 15% of transgender youths participate in sports compared with nearly 70% of all youths nationwide.^26^ Transgender youths are also disproportionately affected by obesity,^27,28^ which has also been increasing over the past decades, especially among adolescents.^29^ Physical activity is an important tool to improve health^30^ and may be particularly important for transgender youths^31,32^ to support their metabolic health, improve bone mineral density, and reduce feelings of loneliness and anxiety. The youths in our study recognized that increasing inclusion and participation of transgender youths could improve both mental and physical health outcomes.^33^

The youths in this study, however, were not without concerns about gender identity–based participation for transgender individuals, and about one-third of respondents felt that restrictive policies should be used. Many of these participants cited concerns about fairness, particularly in female sports, similar to another recent poll^34^; others noted that participation should depend on stage of transition or hormone levels. Respondents frequently mentioned fairness as an impact on cisgender athletes and described how it may affect scholarships and financial opportunities for sports. Prior research has suggested an inherent unfair advantage with identity-based inclusion of transgender athletes in elite sports, especially among transgender women because of testosterone exposure and higher muscle mass.^35^ While there is theoretical evidence of an advantage, limited studies have demonstrated actual inequity related to either an advantage for transgender women or a disadvantage for transgender men.^19^ Efforts to integrate transgender athletes in sports should address perceptions of fairness among cisgender and transgender youths and their parents. Further research into the advantages and disadvantages of identity-based inclusion, beyond the scope of this survey, is needed to support future guidelines and policy development.

The youths in our study reported limited experience participating alongside transgender athletes. This finding is consistent with data on rates of transgender individuals participating in sports.^26,36,37^ With so few transgender athletes, resulting in limited collective playing experiences, the youths in our survey were still able to envision multiple impacts. Many adults, as well as US states and governing bodies in sports and medicine, describe similar perspectives in developing policies or guidelines for and against identity-based participation in sports.^6,36,38^ While some policies, like that of the National Collegiate Athletic Association, aim to preserve “opportunity for transgender student-athletes while balancing fairness, inclusion and safety for all who compete,”^39^ any policy developed without the perspectives of youths risks being not only ineffective but also potentially harmful to youths. Encouraging open discussion among athletes to share their perspectives and experiences, rather than only including adult perspectives, could help youths bring their beliefs and concerns to the table.

### Limitations

This qualitative study is not without limitations. Given the nature of this text-message survey, follow-up questions and probes could not be collected to clarify unclear responses. Additionally, only one-third of respondents (34%) participated in sports and only 5% personally knew someone affected by restrictive policies, which may influence youths’ perceptions and responses.^38^ MyVoice is a large nationwide sample, although it is not nationally representative, which may limit the generalizability of our findings.

## Conclusions

The youths in our study differed in their opinions regarding sports participation among transgender youths but overwhelmingly felt that inclusive policies would affirm and support the mental health of transgender individuals. These findings suggest that increasing affirmation of transgender individuals through outlets like sports could help to mitigate the mental health disparities that exist for transgender individuals. More transgender youths may also choose to participate in sports with inclusive practices and benefit from both mental and physical benefits of physical activity. Negative impacts on fairness were noted by some respondents. Nuanced policies are needed to address transgender athlete participation in competitive sports and should consider the impacts on and perspectives of youths most affected.

## Notes

Supplement.
Data Sharing Statement
