# Supplementary material for: Perspectives of US Youths on Participation of Transgender Individuals in Competitive Sports: A Qualitative Study
Source: JAMA Netw Open. 2023 Feb 8;6(2):e2255107. doi: 10.1001/jamanetworkopen.2022.55107 (PMC9909496; doi:10.1001/jamanetworkopen.2022.55107)
Supplement: Supplement. — Data Sharing Statement [file jamanetwopen-e2255107-s001.pdf]

Data Sharing Statement

Waselewski. Perspectives of US Youths on Participation of Transgender Individuals in Competitive Sports: A Qualitative Study. *JAMA Netw Open*. Published February 08, 2023. doi:10.1001/jamanetworkopen.2022.55107

Data
Data available: No

Additional Information
Explanation for why data not available: Our work uses data from MyVoice (www.hearmyvoicenow.org) a nationwide text message survey of youth (ages 14-24). As part of our IRB approval and protections for this vulnerable population, we require Data Sharing Agreements to be executed with any individual or organization interested in accessing our data. Individuals interested in this data can contact the authors directly to gain access.
